# Advantageous Implications of Reversed Austenite for the Tensile Properties of Super 13Cr Martensitic Stainless Steel

**DOI:** 10.3390/ma15217697

**Published:** 2022-11-01

**Authors:** Peng Wang, Weiwei Zheng, Xinpan Yu, Yanli Wang

**Affiliations:** 1 State Key Laboratory for Advanced Metals and Materials, University of Science and Technology Beijing, Beijing 100083, China; 2 Collaborative Innovation Center of Steel Technology, University of Science and Technology Beijing, Beijing 100083, China

**Keywords:** reversed austenite, super 13Cr martensitic stainless steel, double-step tempering, Kurdjumov–Sachs orientation relationship, phase transformation

## Abstract

The advantageous implications of the microstructure and volume fraction of reversed austenite for the tensile properties of super 13Cr martensitic stainless steel (13Cr SMSS) in an experiment with quenching and double-step tempering treatment in the temperature range of 550–750 °C were investigated. The results show that, with increases in one-step tempering temperature, the content of reversed austenite was enhanced considerably from 0.9% to 13.3%. The reversed austenite distributed in the martensitic lath boundary conformed to the (1$\bar{1}$1)_γ_//(011)_α’_ and [011]_γ_//[$\bar{1}\bar{1}$1]_α’_ Kurdjumov–Sachs orientation relationship with the matrix. When tempered at 675 °C for 3 h for the first stage and 600 °C for 2 h for the second stage, the maximum volume fraction of reversed austenite was approximately 13.3%, achieving uniform elongation of 10.4% and total elongation of 27.2%. Moreover, the product of strength and elongation (PSE) was 23.5 GPa·% higher than other samples. The outstanding combination of high strength and commendable plasticity was due to the phase transformation of the reversed austenite into secondary martensite during tensile straining. The reversed austenite consumed the plastic energy at the tip of the microcrack and made the crack tip blunt, which hindered the further propagation of the crack, consequently increasing the total elongation and improving toughness.

## 1. Introduction

SMSSs have been widely employed in oil fields, forasmuch as the satisfactory combination strategy of strength, ductility and corrosion resistance [1,2]. Tempered martensite and reversed austenite are obtained by the quenching and tempering of SMSS. Reversed austenite is a critical factor in 13Cr SMSS [3,4,5] and improves mechanical properties, especially with excellent low-temperature toughness [6,7]. The formation mechanism of austenite has been liberally studied [8,9,10,11]. Two main mechanisms have been proposed, namely, diffusion shear and the diffusion mechanism [12,13,14]. The partitioning of austenite-forming elements and the morphology and size of prior austenite during heat treatment are the main factors affecting austenite stability [15,16].

The volume fraction of reversed austenite is primarily affected by the tempering technique [17]. Small adjustments in heat treatment parameters will lead to changes in mechanical properties [18]. Zhang et al. have conducted a first-step tempered experiment for different holding times [19]. Song et al. have proven the influence of first-step tempered temperature on tensile properties [12]. The best combination of performance was achieved at 610 °C due to the acquisition of a large amount of reversed austenite. Yan et al. have studied the relationship between reversed austenite volume fractions and mechanical properties [20]. Govindaraj et al. have studied the effect of secondary martensite into which reversed austenite is transformed during deformation [21,22,23,24]. The austenite reversion from lath martensite, a ductile phase, could enhance mechanical behavior through the effects of transformation-induced plasticity (TRIP) [5,25]. Quenching at high temperatures and two-step tempering are required in super martensitic stainless steels, where the volume fraction and stability of reverse austenite can affect the mechanical properties. In practical production, two-step tempering heat treatment is used to increase the content of reversed austenite to acquire satisfactory plasticity and toughness, as demonstrated by the tensile and impact experiments. Though many studies have confirmed the formation mechanism and austenite stability [10,26,27] and their influence on mechanical properties in the related experimental materials [21,28,29], a lot of excellent work has been performed by researchers in related studies. However, the tensile process lacks a comprehensive and in-depth systematic study. The TRIP effect during the tensile process and its influence on the properties of SMSS have not been brilliantly clarified. Additionally, the material under study is ultra-low-carbon super-martensitic stainless steel, and the effect of small differences in composition and heat treatment processes on the volume fraction of austenite is not the same as that of similar materials. Based on the above analysis, tensile tests were carried out to determine the effect of different austenite contents on mechanical properties and the results were compared with related studies. In addition, the tensile interruption test was performed on the specimen with the maximum austenite content, and the variation in austenite content and martensite lath orientation in the phase transformation process under different strain conditions was clearly derived, which is helpful to further understanding of the deformation mechanism and the intrinsic connection of the effect of reversed austenite on the mechanical properties, especially the PSE. The implications of reversed austenite for the tensile behavior of the steel studied in this paper are quite important and must be further studied.

In the present study, the advantageous implications of microstructure and volume fraction of reversed austenite for the tensile properties of 13Cr SMSS subjected to quenching and double-step tempering treatment at temperatures between 550 °C and 750 °C were investigated. The morphology and distribution of reversed austenite were studied by electron back-scattered diffraction (EBSD) and transmission electron microscopy (TEM). The K-S orientation relationship between austenite and martensite was obtained by TEM and transmission Kikuchi diffraction (TKD) technology. The effect of reversed austenite content on tensile performance has been studied using tensile tests and tensile interruption tests. The purpose of this examination was to research the relationship between austenite content and mechanical properties, especially the PSE. The influence mechanism of reversed austenite on tensile performance is discussed, providing evidentiary support for optimizing heat treatment parameters to obtain excellent comprehensive mechanical properties while simultaneously providing a reference for an in-depth understanding of phase transformation and its mechanism during tensile processes.

## 2. Materials and Methods

The as-forged super 13Cr martensitic stainless steel supplied by Taiyuan Iron and Steel Group Co., Ltd in Taiyuan, China, was used in this present work. The chemical composition of 13Cr SMSS, gauged by an Inductively Coupled Plasma Optical Emission Spectrometer (ICP-OES), is shown in Table 1. The as-received steel was cut into blocks with dimensions of 10 mm × 10 mm × 4 mm by Electrical Discharge Machining (EDM) wire cutting; these blocks were used for heat treatment. The specimens were cooled with a coolant to take away heat from the wire cutting process. To avoid the influence on the specimen, the specimen was ultrasonically degreased in acetone and the wire cut marks were abraded with abrasive paper before heat treatment to ensure that the specimen was flat and not affected by the wire cutting. A thin-plate small-sized sample with a gauge length, width and thickness of 10, 3 and 1.4 mm, respectively, was used for tensile and tensile interruption tests. Samples of the same size could be compared and studied. In many tensile-related studies, samples of the same size are used [30].

**Figure 1:**
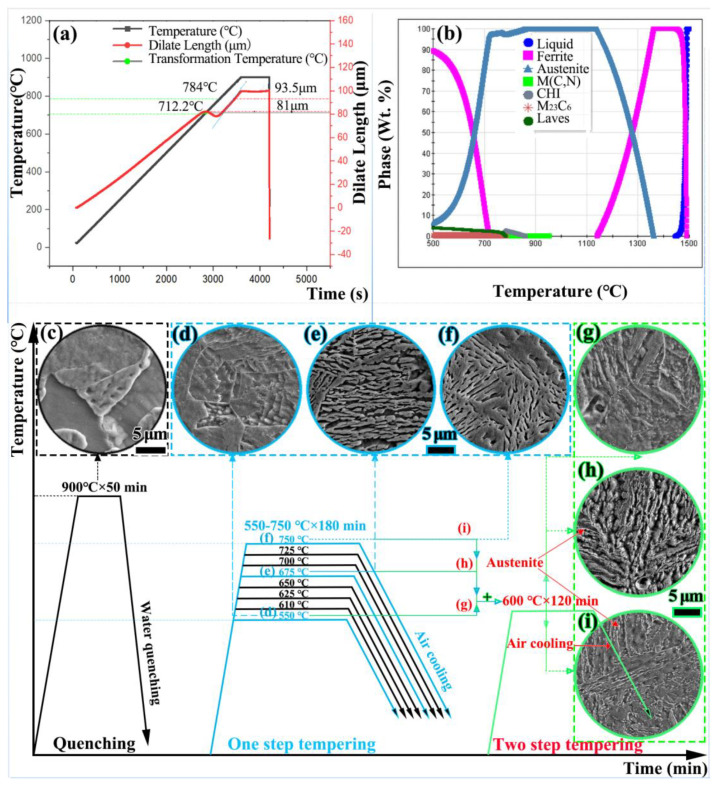
(**a**) Thermal dilatometric curve; (**b**) equilibrium phase diagram; (**c**–**i**) microstructure evolution.

A dilatometer experiment was used to measure phase transformation temperature. Samples with a length of 10 mm and a diameter of 4 mm were machined. The specimen was slowly heated to 900 °C at a rate of 0.25 °C s^−1^. After holding for 600 s at 900 °C, the specimen was quenched to 0 °C at 100 °C s^−1^. The critical temperatures Ac_1_ and Ac_3_ were 712.2 °C and 784 °C, respectively (Figure 1a). Thermo-Calc software was used to calculate and analyze the equilibrium phase composition of the studied steel (Figure 1b), facilitating optimal heat treatment in the corresponding temperature range.

All heat treatments were conducted in air. Quenching was conducted at 900 °C for 50 min, followed by water cooling to environmental temperature. The first stage of tempering was performed from 550 °C to 750 °C for 180 min, followed by air cooling to room temperature. The second stage of the two-step cycle was carried out at 600 °C for 120 min, followed by air cooling to room temperature to increase the reversed austenite content.

The microstructure characteristics of samples were investigated by means of scanning electron microscope (SUPRA55) and TEM (FEI Tecnai G2 F30). Phase analyses were carried out via an XRD technique with Cu Kα radiation with a 2θ range of 40°–120° to verify the phase structure and volume fraction. The quantitative calculation was performed by fitting the diffraction spectrums of {200}_γ_, {220}_γ_, {311}_γ_ and{200}_α’_, {211}_α’_ according to formulas (1) and (2) below [31,32]:

(1) $$V_{\alpha \prime }{+V}_{\gamma }=1$$

(2) $$V_{\gamma }=\frac{1{.4I}_{\gamma }}{1{.4I}_{\gamma }{+I}_{\alpha \prime }}$$

where V_γ_, I_γ_ and I_α’_, V_α’_ are the volume fraction and integrated intensities of reversed austenite and martensite, respectively.

The orientation relationship between austenite and adjacent martensite was confirmed using TEM and TKD. We used EBSD technology to analyze the volume fraction of reversed austenite and the change in orientation of martensitic lath during the tensile process whose condition was set to put off, interruption at engineering strain of 10% and unstrained, respectively.

## 3. Results and Discussion

### 3.1. Microstructures, Phases and Volume Fractions of Reversed Austenite

The microstructure evolution of the as-quenched and double-step tempered specimens are presented in Figure 1c–i. Martensitic packets and laths were coarse in the quenched sample. With increases in first-stage tempering temperature, the tempering martensite lath with a certain orientation gradually became thinner. As shown in Figure 1g–i, more reversed austenite was formed at the martensite lath boundary.

**Figure 2:**
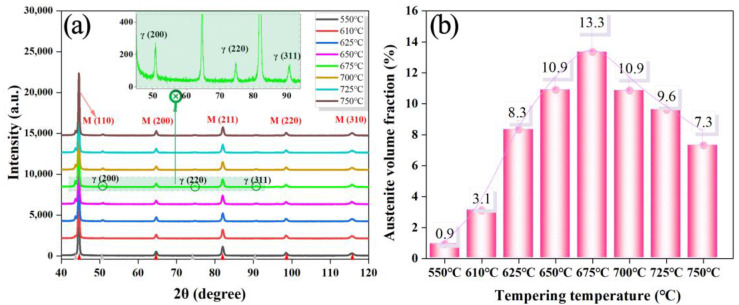
(**a**) X-ray diffraction patterns of the experimental steel treated by quenching and double-step tempering treatment; (**b**) the volume fraction of reversed austenite.

The X-ray diffraction patterns are shown in Figure 2. The (200), (220) and (311) diffraction peaks of the γ phase could be observed in the sample after the first-step tempered treatment at 675 °C for 180 min. The volume fraction of austenite was 13.3 wt.%, demonstrating that the reverse transformation of martensite to austenite occurred at 675 °C. As the tempering temperature increased to 750 °C, the volume fraction of reversed austenite decreased to 7.3 wt.%. The amount of reversed austenite increased with increasing temperature, while the Ni concentration decreased [12].

**Figure 3:**
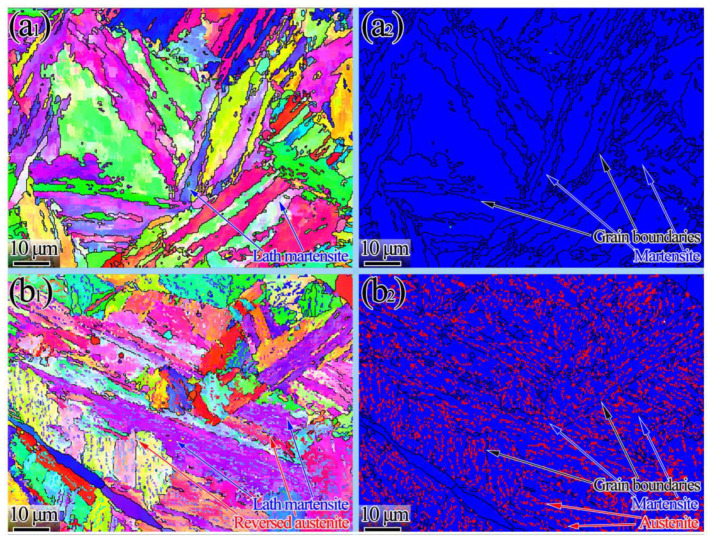
IPF (the high-angle grain boundary marked by black lines (>15°) (the first column) and phase maps (martensite in blue, austenite in red, and black boundaries correspond to misorientation higher than 15°) (the second column)): (**a_1_**,**a_2_**) 675 °C (3 h); (**b_1_**,**b_2_**) 675 °C (3 h) + 600 °C (2 h).

The inverse pole figure (IPF) and phase maps of the samples for the one-step tempering at 675 °C for 180 min and the first stage of double-step tempering at 675 °C for 180 min are shown in Figure 3. They distinctly reveal that there was no austenite on lath martensite (Figure 3a_1_). However, the IPF shows a small variation in the width of the martensite lath for double-step tempering heat treatments. In the double-step tempered specimens, the volume fraction of reversed austenite remarkably increased.

There was almost no reversed austenite in the one-step tempered specimen and the sample consisted of tempered martensite, as shown in Figure 3a_2_. EBSD shows the distribution and content of reversed austenite, shown in Figure 3b_2_; its content was almost identical to the results calculated by XRD (Figure 2b). Phase analysis results show that the reversed austenite was mainly distributed at large-angle grain boundaries and martensite lath boundaries.

**Figure 4:**
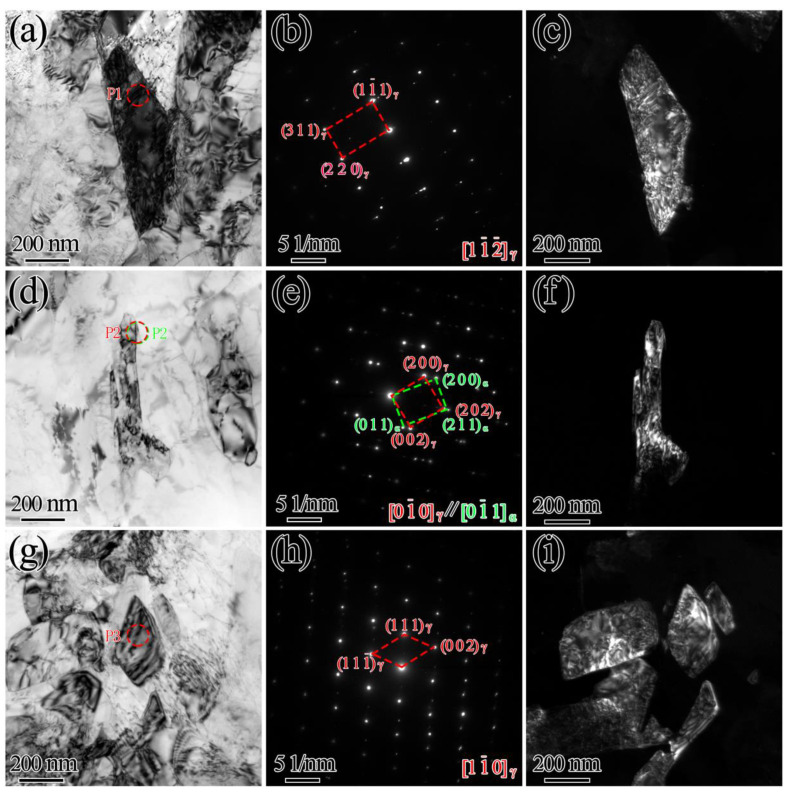
TEM images in double-step tempered samples: (**a**–**c**) 625 °C (3 h) + 600 °C (2 h); (**d**–**f**) 675 °C (3 h) + 600 °C (2 h); (**g**–**i**) 700 °C (3 h) + 600 °C (2 h); (**a**,**d**,**g**) bright-field images; (**b**,**e**,**h**) SAED patterns; (**c**,**f**,**i**) dark-field images.

TEM analysis was performed to study the morphology, size and distribution of reversed austenite. In the bright-field image, the blocky reversed austenite, which can act as an additional obstacle for crack propagation, was distributed along the boundary of martensite lath, as shown in Figure 4. The reversed austenite bore a [0$\bar{1}$0]_γ_//[0$\bar{1}$1]_α_ orientation relationship with the martensite, as shown in Figure 4e. EDS showed Ni and Mn concentrations of approximately 13.6 wt.% and 1.8 wt.%, respectively. More reversed austenite was obtained by two-step tempering, which was used in the steel, promoting the partitioning of Ni and Mn into austenite. The enrichment of Ni and Mn elements in reversed austenite was the main factor leading to reversed austenite stabilization.

The crystallographic orientation of the reversed austenite and martensite was studied utilizing transmission Kikuchi diffraction (TKD) technology for the sample tempered at 675 °C (3 h) + 600 °C (2 h). The reversed austenite phase (denoted by red) is ultimately wrapped by green. The results demonstrate that the martensite phase (marked blue) had good interface distribution characteristics with the reversed austenite phase (marked red), indicating that the two phases were in accordance with the specific orientation relationship of {111}_γ_//{011}_α’_ and <011>_γ_//<111>_α’_.

**Figure 5:**
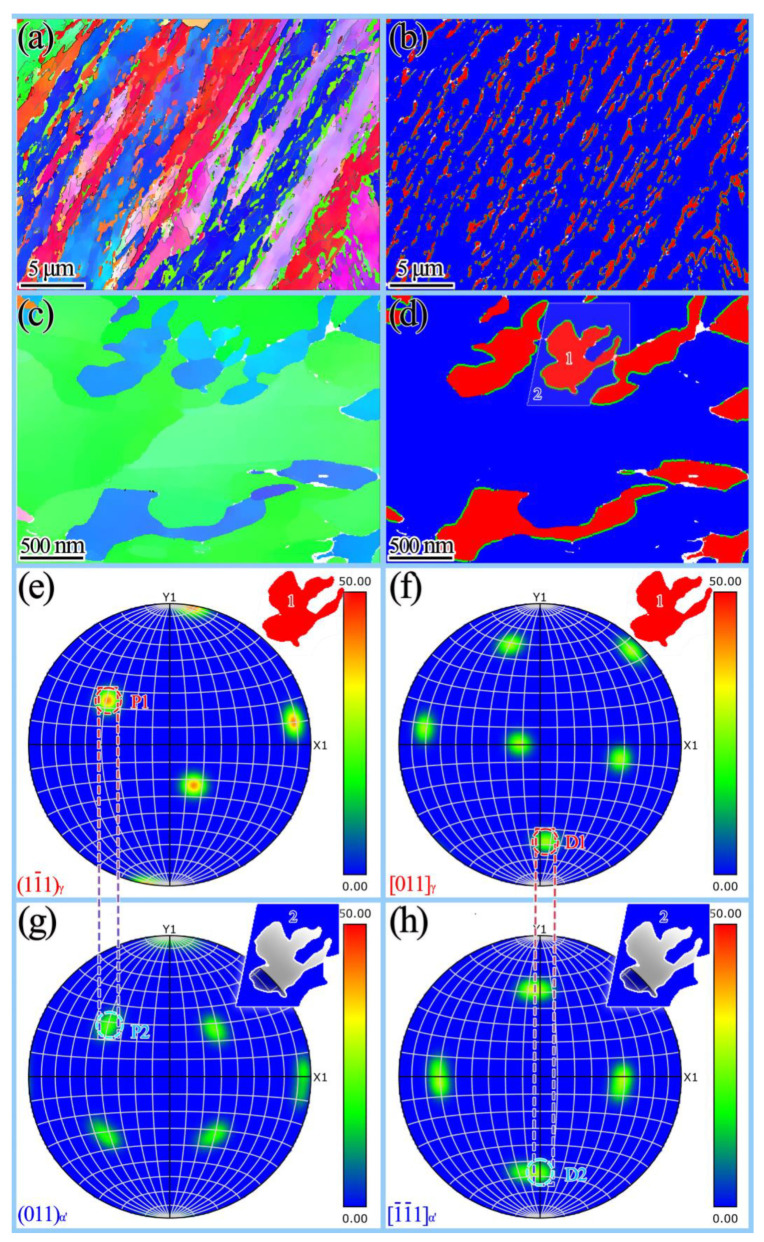
Crystallographic orientation relationship assay of two phases by TKD for the sample tempered at 675 °C (3 h) + 600 °C (2 h): (**a**) IPF; (**b**) K-S orientation relationship of the martensite and reversed austenite phase (martensite in blue and austenite in red); (**c**) IPF (high amplification); (**d**) K-S orientation relationship of the martensite and reversed austenite phase (high amplification, martensite in blue and austenite in red); (**e**–**h**) the pole figures of reversed austenite and martensite.

To further elucidate the orientation relationship of reversed austenite and martensite, the sample was amplified 100,000 times by TKD to obtain finer microstructure at a higher resolution (Figure 5c,d). The polar figure characterizing the orientation relationship of two phases was obtained by taking the adjacent two phases at positions 1 and 2 in Figure 5d. It can be observed that martensite and reversed austenite met the (1$\bar{1}$1)_γ_//(011)_α’_ and [011]_γ_//[$\bar{1}\bar{1}$1]_α’_ K-S orientation relationship. The orientation relationship reduced the interfacial energy of reversed austenite nucleation [12]. Wang et al. [33] have shown that the orientation relationship plays a crucial role in the stability of reversed austenite.

### 3.2. Effect of Reversed Austenite on Tensile Properties

**Figure 6:**
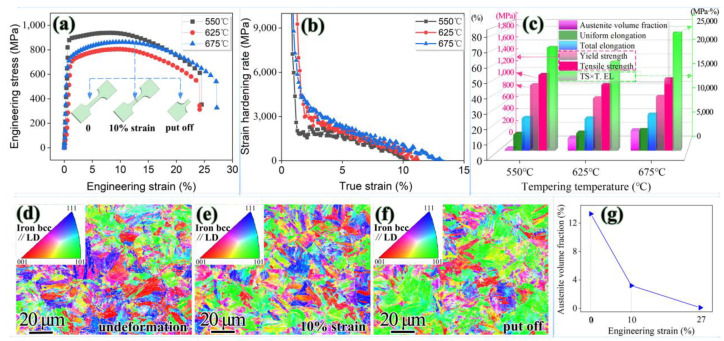
(**a**) The tensile engineering stress–strain curves; (**b**) strain hardening rate curves; (**c**) The value extracted from tensile curves; (**d**–**f**) inverse pole figure of martensitic; (**g**) volume fraction of reversed austenite obtained via putting off, interruption at 10% strain and unstrained, respectively.

Figure 6 shows the tensile test results for first-stage tempering at 550 °C, 625 °C and 675 °C for 3 h, respectively. These three samples are named sample 1, sample 2 and sample 3, respectively. Strength, elongation and PSE as a function of the reversed austenite volume fraction are shown in Figure 6c. The maximum volume fraction of reversed austenite was 13.3% for sample 3, achieving uniform elongation of 10.4% and total elongation of 27.2%. Moreover, PSE was 23.5 GPa·% higher than other samples (Figure 6c and Table 2). In addition, the sample showed a striking ability to sustain a high strain hardening rate over a wide strain region, which was a prerequisite for attractive ductility (Figure 6b). This indicated that the existence of the reversed austenite had a good effect on the ductility. The experimental results are consistent with the effect of austenite on mechanical properties in related materials [12,34].

The variation rule of the volume fraction of reversed austenite for sample 3 as a function of engineering strain is shown in Figure 6g. With tensile progression, the volume fraction of reversed austenite decreased monotonously with the increase in engineering strain. Surprisingly, the yield strength did not conform to the opposite trend with the volume fraction of austenite. During the tensile process, the orientation along the tensile direction gradually paralleled to <110>, shown in Figure 6d–f, and the reversed austenite transformed into new martensite, shown in Figure 6g. Reversed austenite with lower mechanical stability was more sensitive to the increasing strain. The morphology, size and stability of reversed austenite were different under different heat treatment conditions, so the volume fraction of austenite transformation was different under the same strain. Austenite stability affected the TIRP effect, resulting in differences in mechanical properties [35,36,37]. The three strain values shown in Figure 6g correspond to three stages of the tensile curve, shown in Figure 6a. The initial strain was small, corresponding to the elastic phase of the tensile curve, where austenite had barely begun to transform. The yield strength of samples 2 and 3 was almost the same and both were lower than that of sample 1, which conformed to the traditional cognitive. When the engineering strain was gradually increased to 10%, the TRIP effect played a strengthening role. Therefore, compared with sample 2, sample 3 had higher strength and stronger resistance to deformation in the strengthening stage. When the strain was greater than 10%, sample 3, with the most austenite, still had the TRIP effect. Strength and elongation in the necking stage were greater than those of samples 1 and 2 [33].

The excellent combination of high strength and good plasticity improved due to secondary martensite formation assisted by the austenite reversion process and a striking ability to sustain a high strain hardening rate over a wide strain region. Compared to samples 1 and 2, sample 3 exhibited a superior maximum tensile work hardening, which is in agreement with the result that the deformation-induced martensite transformation remarkably increased work hardening. This characteristic has been frequently reported in the relative literature on work-hardening behavior [38,39].

**Figure 7:**
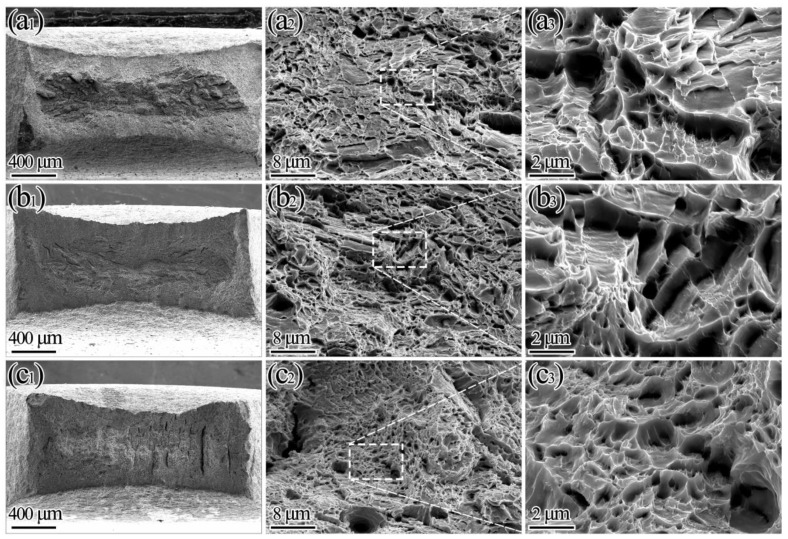
Overall macroscopic (first column), microstructure (second column) and local amplification (third column) of fracture morphology analysis of different tempering temperatures: (**a_1_**–**a_3_**) 550 °C (3 h) + 600 °C (2 h); (**b_1_**–**b_3_**) 625 °C (3 h) + 600 °C (2 h); (**c_1_**–**c_3_**) 675 °C (3 h) + 600 °C (2 h).

Fracture morphology analysis of different tempering temperatures is shown in Figure 7. Different tempering temperatures lead to different volume fractions of reversed austenite, resulting in differences in fracture morphology, which can objectively reflect the level of strength and plasticity. After undergoing tempering at 675 °C (3 h) + 600 °C (2 h), the sample had a better PSE that was 23.5 GPa·% higher than other samples, achieving uniform elongation of 10.4% and total elongation of 27.2%. The increase in elongation was confirmed by SEM observation of fracture morphology; toughness characteristic dimples are fairly shown in Figure 7c_3_. The larger the dimple size (diameter and depth), the better the plasticity of the material. These dimples may generate from the interfaces between the fresh martensite and martensite matrix due to strength differences [24,40,41]. The plasticity of the sample tempering at 675 °C (3 h) + 600 °C (2 h) significantly improved, showing the maximum volume fraction of reversed austenite. In the process of tensile and fracture, the reversed austenite hindered crack propagation and it was necessary to continuously consume energy, causing significant plastic deformation.

**Figure 8:**
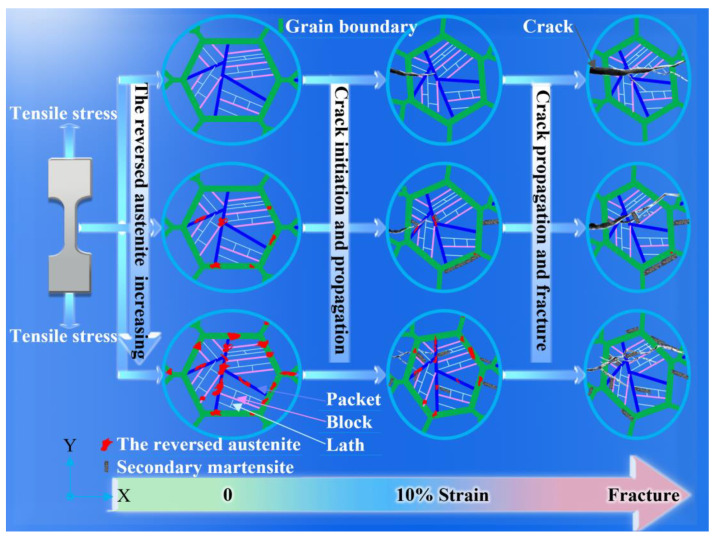
The schematic diagram of the mechanisms governing the effect of reversed austenite on strength and elongation.

The sample, tempering at 675 °C (3 h) + 600 °C (2 h), possessed excellent toughness due to a higher austenite content. The essential reason for the higher PSE was the TRIP effect [42]. The internal mechanism and influence law are shown in Figure 8. In the tensile process, the reversed austenite transformed into secondary martensite. The reason for the work hardening and strength improvement was the non-uniformity of plasticity [43].

The higher the volume fraction of austenite, the more secondary martensite transformed. The gradual transformation of austenite into a great amount of secondary martensite was acquired at a superior strain lever and the effect of TRIP on strength became more pronounced.

As shown in the schematic diagram in Figure 8, fine reversed austenite played an obvious role in tensile deformation. When the crack propagated to the reversed austenite phase, the austenite consumed a large amount of deformation work and made the crack tip blunt, which hindered the further propagation of the crack and thus increased the total elongation and toughness. TRIP effect, consuming the plastic energy at the tip of the microcrack [44], alleviated local stress concentration and hindered crack initiation and propagation [45].

Sample 3 possessed the maximum volume fraction of reversed austenite and showed an obvious improvement in total elongation and PSE values, which stemmed from the continuable TRIP effect and high strain hardening rate (Figure 6b). Additionally, the non-uniform elongation of sample 3 was obviously larger than that of samples 1 and 2, which may indicate a better resistance to crack propagation. There are consistent conclusions concerning quenched and partitioned (Q&P) steel that support and corroborate one another [24]. In brief, the TRIP effect, consuming a large amount of deformation work, alleviated local stress concentration. The reversed austenite consumed the plastic energy at the tip of the microcrack and made the crack tip blunt, which hindered the further propagation of the crack, consequently increasing the total elongation and improving toughness.

## 4. Conclusions

The microstructure evolution, mechanical properties and deformation behavior were investigated in a 13Cr SMSS subjected to one-step and two-step tempering processes. Advantageous implications of the reversed austenite for the tensile properties of 13Cr SMSS were discussed. The mechanism whereby reversed austenite is beneficial to strength and toughness was elucidated. The main findings and conclusions are as follows:

1. Reversed austenite is distributed along the boundary of martensite lath and bears the (1$\bar{1}$1)_γ_//(011)_α’_ and [011]_γ_//[$\bar{1}\bar{1}$1]_α’_ Kurdjumov–Sachs (K–S) orientation relationship with the martensite.
2. When tempered at 675 °C for 3 h for the first stage and 600 °C for 2 h for the second stage, the maximum volume fraction of reversed austenite is approximately 13.3%, achieving uniform elongation of 10.4% and total elongation of 27.2%. Moreover, the product of strength and elongation is 23.5 GPa·% higher than other samples.
3. The outstanding combination of high strength and commendable plasticity has been achieved due to the secondary martensite formation assisted by the austenite reversion process and a striking ability to sustain a high strain hardening rate over a wide strain region.
4. The TRIP effect, consuming a large amount of deformation work, alleviates local stress concentration. The reversed austenite consumes the plastic energy at the tip of the microcrack and makes the crack tip blunt, which hinders the further propagation of the crack, consequently increasing the total elongation and improving toughness.

## Figures and Tables

**Table 1:** Chemical composition of SMSS (wt.%).

C	Cr	Ni	Mo	Mn	Al	V	Si	P	S	Fe
0.01	13.2	5.06	1.98	0.66	0.015	0.07	0.25	0.0065	0.0064	Bal.

**Table 2:** The austenite volume fraction and mechanical properties of samples 1, 2 and 3.

No.	Austenite Volume Fraction (%)	Yield Strength (MPa)	Ultimate Tensile Strength (MPa)	Uniform Elongation (%)	Total Elongation (%)	PSE GPa•%
Sample 1	0.9	881.2	938.6	8.5	24.1	22.6
Sample 2	8.3	714.7	803.8	10.3	23.7	19.1
Sample 3	13.3	721	862.5	10.4	27.2	23.5

## Data Availability

The raw/processed data required to reproduce these findings cannot be shared at this time as the data also forms part of an ongoing study.
